# Spinal subdural hematoma: A report of 3 cases related to antiplatelet agent use and traumatic compression fracture

**DOI:** 10.1016/j.radcr.2021.03.041

**Published:** 2021-04-13

**Authors:** Bo-mi Kim, Min-Yung Chang, Seung Hyun Lee, Joong Won Ha

**Affiliations:** Department of Radiology Orthopedic surgery, National Health Insurance Service Ilsan Hospital, Goyang, Republic of Korea

**Keywords:** Spinal subdural hematoma, Ant-platelet agent, Traumatic compression fracture

## Abstract

Spinal subdural hematoma is a rare condition presenting with symptoms of back pain and neurologic deficits. The etiology is largely idiopathic, followed by anti–coagulant use and vascular malformation. Traumatic subdural hematomas associated with compression fractures are rare, with only a few old case reports. Magnetic resonance imaging is the modality of choice for the diagnosis of spinal subdural hematoma. Treatment is surgical decompression when neurologic deficits exist; however, conservative management is a good option in patients without neurologic symptoms with reported spontaneous hematoma regression. Herein, we report 3 cases of spinal subdural hematoma, 2 spontaneous cases related to anti–platelet agent use and 1 with acute traumatic compression fracture. T1-weighted fat-saturated images clearly showed the hematoma and increased the confidence level of the diagnosis. In summary, we suggest that magnetic resonance imaging can clearly visualize the spinal subdural hematoma and is excellent for diagnosis and follow up. Anti–platelet agent use and compression fracture are probable etiologies of spinal subdural hematoma.

## Introduction

Spinal subdural hematoma is a rare condition with a reported incidence of 4.1% of all spinal hematomas [1]. The etiology of a spinal hematoma is largely idiopathic (approximately 30% cases), followed by anti–coagulant therapy and vascular malformations [1]. The most common symptoms are back pain and neurologic deficits related to the location of nerve compression. Magnetic resonance imaging (MRI) is the modality of choice for the detection and diagnosis of spinal hematoma. Subdural hematoma is diagnosed when the hematoma is located in the subdural space, preserving the epidural fat plane without displacement of the dura mater [2]. In patients with cord compression or cauda equine syndrome, emergency decompression and hematoma evacuation are mandatory. However, if there is no neurologic deficit, conservative management with MRI follow up may be recommended since some hematomas resolve spontaneously [3,4]. Herein, we report 3 cases of spinal subdural hematoma; 2 related to anti-platelet agent use, and 1 with a traumatic compression fracture.

**Fig. 1:**
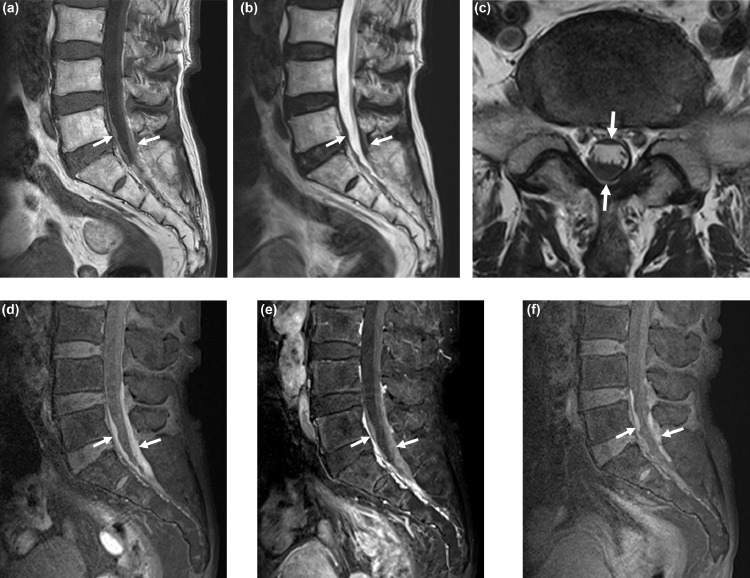
An 83-year-old man with subacute spinal subdural hematoma. (A and B) The sagittal T1-weighted (A) and T2-weighted (B) images show hyperintensity and hypo-intensity of the subdural hematoma (arrows), respectively. (C) The axial T2-weighted image confirming the subdural location of the hematoma (arrows). (D and E) Sagittal fat-saturated T1-weighted images without (D) and with (E) enhancement show hyperintensity of the subdural hematoma (arrows). The fat-saturated T1-weighted image visualizes the extent of the hematoma more clearly than conventional T1- and T2-weighted images. (F) Sagittal fat-saturated T1-weighted magnetic resonance image at the 1-month follow up shows markedly decreased volume of the hematoma (arrows).

**Fig. 2:**
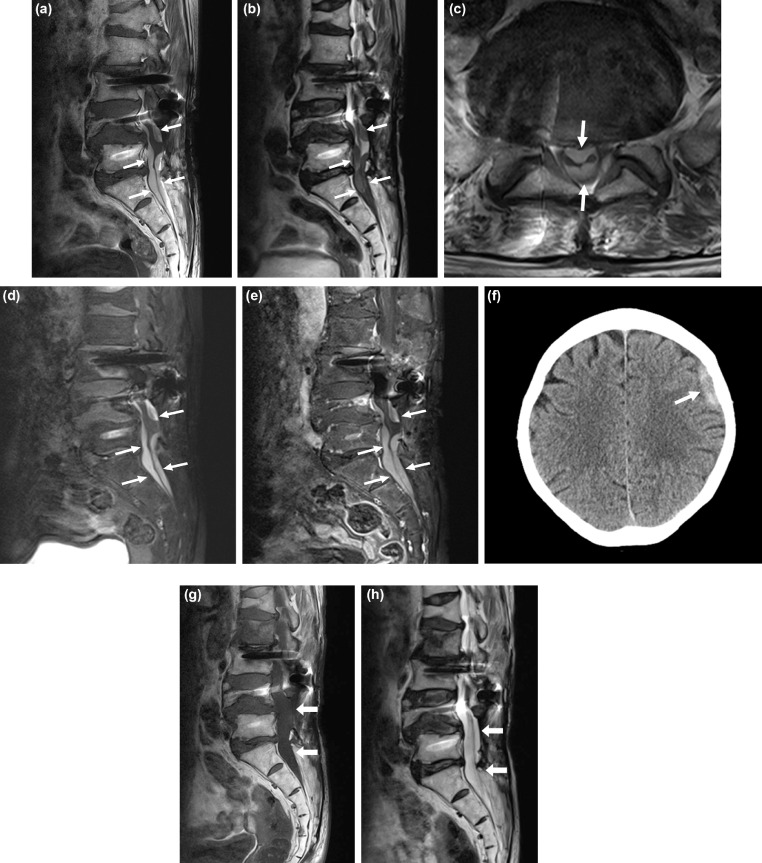
A 79-year-old woman with subacute spinal subdural hematoma and intracranial subdural hematoma. A and B. The sagittal T1-weighted (A) and T2-weighted (B) images show hyperintensity and hypointensity, respectively, of the subdural hematoma (arrows). Metal artifacts related to previous lumbar fusion and old compression fractures at the L1 and L4 are also seen. (C) The axial T1-weighted image confirming the subdural location of the hematoma (arrows). (D and E) Sagittal fat-saturated T1-weighted images without (D) and with (E) enhancement show hyperintensity of the hematoma (arrow). (F) Axial brain computed tomography (CT) image showing acute subdural hematoma on left convexity (arrow). (G and H) The sagittal T1-weighted (G) and T2-weighted (H) images show that the subdural hematoma had decreased in size remarkably after 10 months (arrows).

**Fig. 3:**
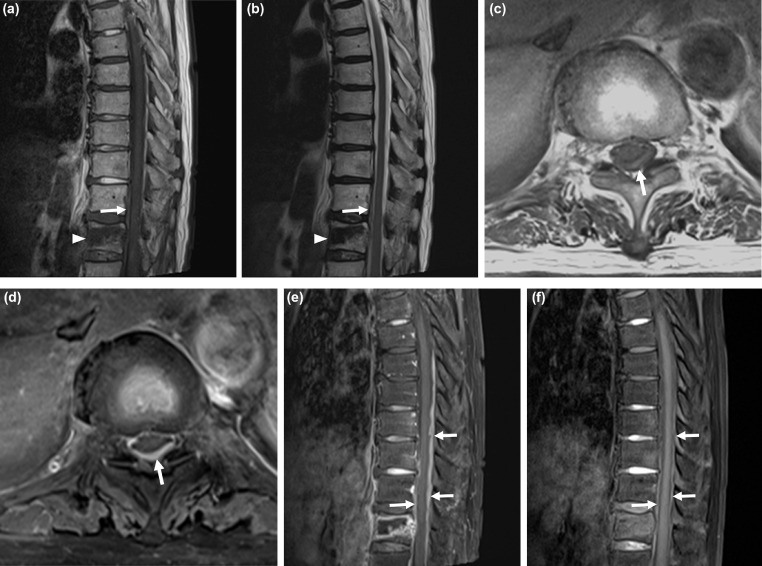
An 89-year-old woman with acute compression fracture and associated acute spinal subdural hematoma. (A and B) The sagittal T1-weighted (A) and T2-weighted (B) images show iso- to slight hyperintensity and hyperintensity, respectively, of the subdural hematoma. The acute subdural hematoma is indistinct from the cerebrospinal fluid (CSF) on both images (arrow). Acute T12 compression fracture is also seen (arrowhead). (C) The axial T1-weighted image confirming the subdural location of the hematoma (arrow). (D and E) The axial (D) and sagittal (E) fat-saturated enhanced T1-weighted images show hyperintensity of the hematoma (arrows). (F) The sagittal non-enhanced fat-saturated T1-weighted image taken 3 days later shows a slight decrease in size of the subdural hematoma. (arrows).

## Case report

### Case 1

An 83-year-old man was referred to our radiology department for lumbar spine MRI due to sudden onset sacral pain without trauma that persisted for 3 days. He had underlying coronary artery occlusive disease (status post stent insertion), hypertension, and diabetes mellitus and was on medication for the same. He was taking aspirin 100 mg/day without any anticoagulant therapy. Radiography showed only a mild degree of lumbar spondylosis. MRI revealed loculated fluid collection within the thecal sac from the L4-S2 level, which was hyperintense on T1-weighed images and hypo-intense on T2-weighted images. On enhancement images, the lesion showed hyperintensity. T1 fat saturation images were obtained to exclude an enhancing soft tissue lesion suggesting a tumor; however, the lesion still showed hyperintensity (Fig. 1). A diagnosis of spinal subdural hematoma was made. No other lesions that could cause sacral pain were detected on the MRI. Initial laboratory tests, including a coagulopathy panel, showed no remarkable abnormality. Treatment included conservative management with pain control because there was no neurologic deficit. Follow up MRI was performed 1 month later, and the subdural hematoma had decreased in size remarkably. Sacral pain improved significantly.

### Case 2

A 79-year-old woman presented with persistent lower back pain after undergoing L3-L4-L5 fusion surgery 1½years ago. The pain had been aggravated recently, and bilateral lower extremity weakness had developed since the last 3 weeks. She had hypertension and was on medication with clopidogrel bisulphate (75 mg/day tablets). Motor weakness was estimated to be normal on physical examination (grade 5/5). Radiography did not show hardware-related complications, but revealed adjacent segment disease at the L2/3 level. On MRI, subdural fluid collection, with hyperintensity on T1-weighted images and hypo-intensity on T2-weighted images, was noted from the L4 to the S2 level. The lesion was hyperintense on both enhanced T1 and T1 fat-saturated images (Fig. 2A-E). Additionally, the MRI showed L2/3 moderate central canal stenosis as adjacent segment disease. MRI of the brain was also performed on the same day because she had a history of minor head trauma (she had slipped and bumped her head against a wall). MRI revealed a subdural hematoma at the left convexity (Fig. 2F). Laboratory test results were within normal limits. The plaque was due to spinal and intracranial hematoma formation. She received conservative treatment with pain management. Computed tomography of the brain was performed the next day and revealed that the intracranial subdural hematoma had decreased slightly. Follow up spinal MRI was performed 10 month later, and the subdural hematoma had decreased in size remarkably (Fig. 2G and H). Lower back pain also improved gradually.

### Case 3

An 89-year-old woman visited our emergency department due to lower back pain after a slip-down injury. She was a known hypertensive and on medication for the same. T12 and L3 compression fractures were detected on radiography, and MRI was performed, which revealed acute compression fracture at T12 and subdural hematoma along the T7 to the T12 level. The subdural hematoma showed iso- to slight hyperintensity on T1-weighted images and hyperintensity on T2-weighted and enhanced images. Iso- to slight hyperintensity on T1-weighted fat-saturated images and hyperintensity on T2-weighted images indicated that the lesion was an acute hematoma (Fig. 3A-E). Laboratory test results were unremarkable. She was advised complete bed rest and pain control for the acute compression fracture. Surgical intervention was withheld because she experienced no neurologic deficit. On a follow up MRI performed 3 days later, the subdural hematoma showed a slight decrease in size (Fig. 3F). At the 1-month follow-up in the outpatient clinic, her pain had improved significantly, and radiography revealed no progressive height loss of the compression fracture.

## Discussion

In all 3 cases, the MRI showed findings of spinal subdural hematomas. Although previous studies have reported underlying coagulopathy or anti–coagulant medication as the second contributing cause, none of our patients were on anti–coagulant therapy or had abnormal coagulation profiles. All patients were on medication for hypertension, and 2 of them were on anti–platelet medication. A previously reported case of spinal subdural hematoma also mentioned that the patient was on anti–platelet therapy [5]. In the present case 1, there was no history of trauma, and spontaneous spinal subdural hematoma was the only finding on MRI that could explain the new-onset lower back pain. Similar to other cases of spontaneously resolving subdural hematomas, the subdural hematoma in this case regressed rapidly. In case 2, the patient had a minor head trauma, and both intracranial and spinal subdural hematomas were noted on MRI. Several previous reports have shown the co-existence of cranial and spinal subdural hematomas [5], [6], [7], [8]. There are 2 hypotheses explaining this concomitance: first, intracranial hematoma is re-distributed to the dependent portion, and second, increased intracranial subdural pressure increases the shearing force between the subarachnoid and subdural spaces and induces spinal subdural hematoma [4]. In case 3, acute compression fracture and continuous subdural hematoma were diagnosed. To the best of our knowledge, there are no published studies describing compression fractures and associated subdural hematomas. Donald et al. reported 2 cases of subdural hematoma associated with a compression fracture [9]. Most of the previously reported traumatic spinal subdural hematoma cases had a history of head trauma rather than that of spinal trauma [5,^8^,10]. Even though compression fracture-related epidural hematoma is common, spinal subdural hematoma is a very rare entity, and we hypothesize that injury to the dura itself can lead to the formation of a subdural hematoma. In the present cases, MRI played an important role in the diagnosis and follow up. T1-weighted fat-saturated images clearly described the extent of the lesion and confirmed the diagnosis.

In conclusion, MRI clearly visualizes the spinal subdural hematoma and is excellent for diagnosis and follow up. Antiplatelet agent use and compression fracture are probable etiologies of spinal subdural hematoma.
